# Determinants of Insulin Requirement in Children With Type 1 Diabetes Mellitus: Experience From a Tertiary Care Center in North-Western India

**DOI:** 10.7759/cureus.105477

**Published:** 2026-03-19

**Authors:** Preeti Sharma, Varuna Vyas, Abhishek Kothari, Vikas Yadav, Akhil Dhanesh Goel, Kuldeep Singh

**Affiliations:** 1 Pediatrics, All India Institute of Medical Sciences, Jodhpur, Jodhpur, IND; 2 Pediatric Endocrinology, Pandit Deendayal Upadhyaya Medical College, Churu, IND; 3 Community Medicine & Family Medicine, All India Institute of Medical Sciences, Jodhpur, Jodhpur, IND

**Keywords:** celiac disease, hypothyroidism, insulin titration, maximum insulin requirement, type 1 diabetes mellitus

## Abstract

Objectives

Insulin, the main drug used in the treatment of Type 1 Diabetes Mellitus (T1DM), has a variable dose requirement and often needs individualized titration in pediatric patients. This individualization of treatment can be time-consuming and challenging. We conducted this study to analyze insulin requirement in children with T1DM and its correlation with various patient variables.

Methods

The cross-sectional ambispective study included pediatric patients with T1DM aged <18 years who were admitted to the department of pediatrics at our hospital from November 2022 to October 2023. Insulin titration was tailored to individual needs during admission. Maximum insulin requirement during admission (IU/kg/day) and insulin requirement at discharge (IU/kg/day) were recorded along with relevant demographic and clinical details in a pre-prepared proforma. A total of 64 patients were included, with a male-to-female ratio of 1.55:1. Ten patients were studied retrospectively, and 54 patients were analyzed prospectively.

Results

The maximum insulin requirement in terms of IU/kg/day during hospitalization was significantly higher among children below 5 years of age, female patients, and those presenting with celiac disease or diabetic ketoacidosis (DKA) (p < 0.05). Insulin requirements at discharge were notably higher in children with celiac disease (P = 0.031), while no significant disparities were observed based on age, sex, presence of DKA, or hypothyroidism. Linear regression analysis identified age, sex, presence of DKA, and maximum insulin dose required during hospitalization as significant predictors of insulin dose at discharge (R2 = 0.92, p < 0.001).

Conclusions

These findings may provide valuable insights for refining insulin titration and discharge planning. Further studies are imperative in developing nations due to limited data on pediatric insulin requirements.

## Introduction

Type 1 Diabetes Mellitus (T1DM) is a syndrome of disturbed energy metabolism involving carbohydrates, proteins, and fats, caused by an absolute deficiency of endogenous insulin secretion. These metabolic disturbances lead to various microvascular and macrovascular complications like diabetic nephropathy, diabetic retinopathy, and diabetic neuropathy. Insulin has been the life-saving treatment for diabetes since 1921. Near normoglycemia has been well established as a treatment goal for diabetes based on the results of the landmark Diabetes Control and Complications Trial (DCCT) [1-3]. However, achieving normoglycemia is challenging because there is no predictable treatment dose or regimen that applies to all individuals. This becomes more challenging in pediatric patients with T1DM.

There is no consensus on the optimal insulin starting dose in children with new-onset T1DM. According to the International Society for Pediatric and Adolescent Diabetes (ISPAD) clinical consensus practice guidelines 2022, the starting dose for prepubertal children ranges from 0.7 to 1.0 IU/kg/day [4]. The American Association of Clinical Endocrinologists and American College of Endocrinology (AACE/ACE) guidelines recommend a starting dose of insulin ranging from 0.4 to 0.5 IU/kg/day. In contrast, the American Diabetes Association (ADA) recommends that the initial total daily dose (TDD) for children and adolescents range from 0.5 to 1.0 IU/kg [5-6]. The insulin requirement in children and adolescents with T1DM is dynamic due to ongoing growth, development, and hormonal changes, necessitating frequent dose adjustments [4].

In developing and resource-limited countries, where there are few dedicated pediatric endocrinologists and diabetes educators, initiating and accurately titrating a drug that requires frequent monitoring is difficult. Application of guidelines from developed countries may not always be appropriate due to differences in body composition, dietary habits, insulin type, and insulin delivery method. We have therefore evaluated the total daily insulin requirement in pediatric patients with T1DM in the northwestern Indian population and the factors influencing it.

## Materials and methods

We conducted a hospital-based ambispective observational study in the Department of Pediatrics at the All India Institute of Medical Sciences (AIIMS), Jodhpur, India. The study was started after obtaining permission from the institute’s ethics committee (approval no. AIIMS/IEC/2023/4293).

During the study period (November 2022 to October 2023), a total of 66 patients were evaluated for enrollment. All children admitted during the study period with T1DM (both newly diagnosed and previously followed-up cases) aged less than 18 years were included. Two were excluded based on repeat admissions. Data were collected retrospectively from inpatient records for 10 patients and prospectively in the ward for 54 patients by a single investigator and analyzed after proper verification. Diagnosis of T1DM was made based on the clinical presentation, as defined in the ISPAD guidelines 2022 [4]. Children on corticosteroids or with suspected type 2 diabetes mellitus (T2DM) or suspected monogenic forms of diabetes mellitus were excluded based on clinical criteria defined under ISPAD guidelines 2022 and genetic evaluation in cases of monogenic diabetes [4]. No intervention was done for the study. Two insulin dosing regimens were used during insulin titration in admitted patients as per patient or family preference, with affordability as the main factor. Both regimens are described in detail as follows.

Basal-bolus regimen

Fifty-two patients used this insulin regimen. Starting dose of insulin was prescribed according to ISPAD guidelines 2022 with 30-50% of total daily requirement as basal insulin in the form of long-acting insulin, glargine, and the rest in the form of prandial insulin as rapid-acting or short-acting insulin (aspart/regular insulin) by either pen or syringe and vial as per patient preference. Seven-point glucose monitoring was performed (three times premeal, three times 2 hours postmeal, and once after midnight, around 2 AM). During hospitalization, along with blood glucose optimization, the child with T1DM and his family were educated regarding diet, diabetes care, insulin administration, and home blood glucose monitoring. Subsequent insulin doses were titrated as per the patient’s blood glucose levels. Patients were discharged when they were confident in their diabetes management at home and when at least 70% of blood glucose readings were between 70-180 mg/dl, with no more than one episode of hypoglycemia or symptomatic hypoglycemia in the last 24 hours [4]. At discharge, a 10-20% reduction in the total insulin dose was performed, as the child was likely to become more ambulatory in household settings to avoid hypoglycemia.

Split mix regimen

The majority of patients used only a basal-bolus regimen, but 12 patients used the split mix regimen due to affordability. Intermediate-acting insulin, neutral protamine Hagedorn (NPH), is used as basal insulin, given twice daily (before breakfast and before dinner), and short-acting insulin, regular insulin, is administered three times premeal as prandial insulin by syringe. Rest, insulin dose titration, and discharge criteria are the same as in the basal-bolus regimen described earlier. If the patient was on continuous glucose monitoring (CGM), the dose required to maintain blood glucose levels in range 70% of the time was considered [4]. We were able to use CGM in only two patients due to cost constraints.

Demographic profile of patients, discharge dose of insulin in terms of International units of insulin per kilogram body weight per day (IU/kg/day), maximum insulin dose required during hospital stay (IU/kg/day), duration of hospital stay, associated comorbidities like thyroid status, celiac disease, and presence of diabetic ketoacidosis (DKA) at admission as per ISPAD criteria 2022 and glycated hemoglobin (HbA1c) levels were recorded in a pre-designed proforma [4]. Newly diagnosed cases of celiac disease and hypothyroidism, based on anti-tissue transglutaminase antibody levels (anti-tTg) and thyroid-stimulating hormone (TSH), done as per laboratory cut-off values with good treatment compliance, were included. All the patients were diagnosed with celiac disease during this hospitalization and initiated on a gluten-free diet only at the time of enrollment in the study. 

Written informed consent was obtained from parents, and age-appropriate assent was taken from the children.

Statistical analysis

Normality of the data was assessed by the Kolmogorov-Smirnov test. Categorical variables are presented as frequencies and percentages. Continuous variables are presented as median (Interquartile range, IQR). Nonparametric statistical tests were used because the data were skewed. The Mann-Whitney U test was used to assess differences between two groups. The Kruskal-Wallis H test was used to test differences between variables with >2 groups. For comparisons, the test statistics along with corresponding p-values were reported. Box-and-whisker plots were used to represent insulin requirements across groups. The association between the discharge insulin dose and several factors was assessed by linear regression, and the best-fit model was selected. Analysis was done using the SPSS statistical software v.26 (IBM Corp., Armonk, USA).

## Results

**Figure 1:**
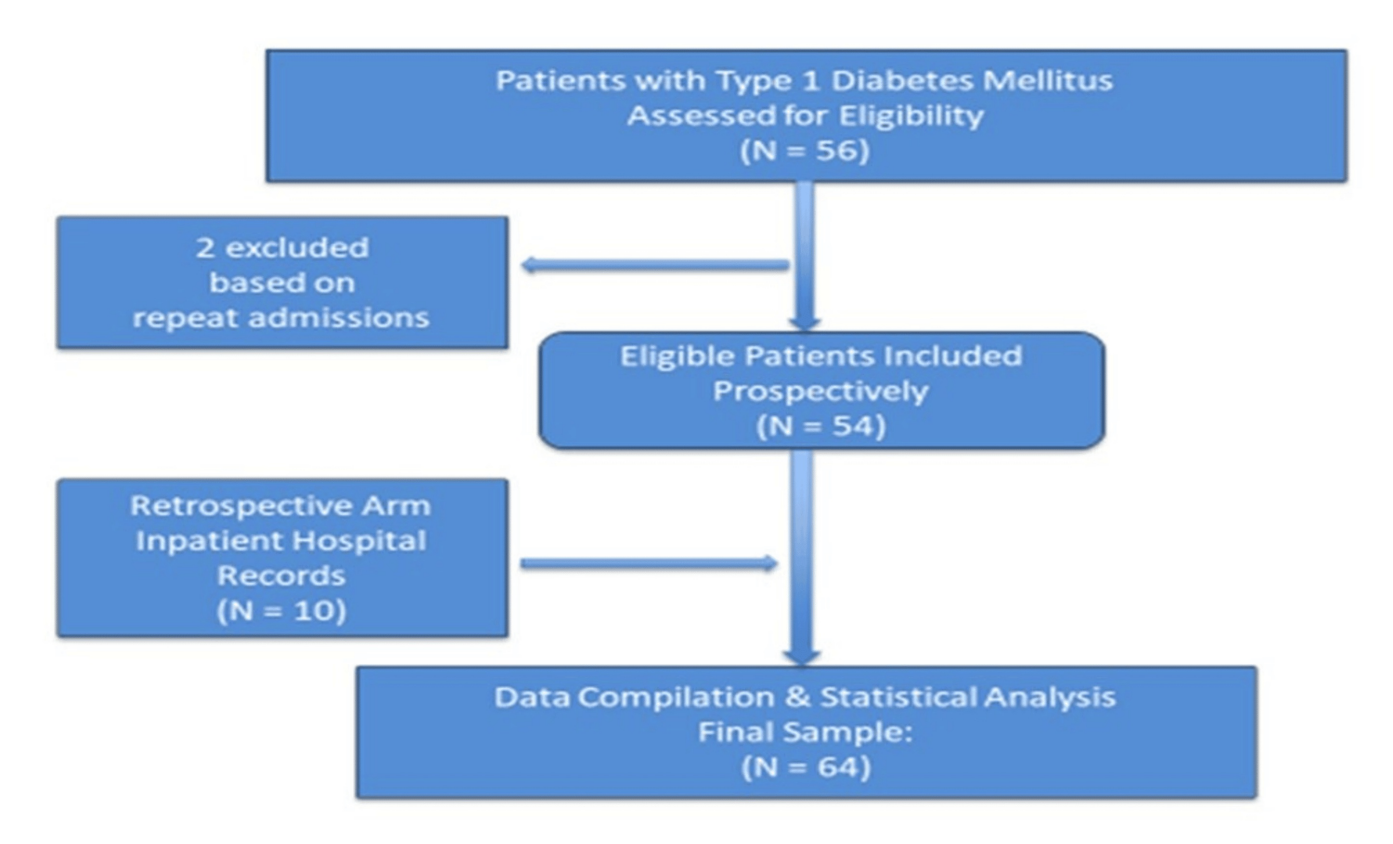
Flow diagram of the study methodology

We included 64 children with T1DM in our study (Figure 1). Among the study population, the majority (39, 60.9%) were males. Seventeen patients were below 5 years of age (Males = 09, Females = 08), 17 were between 6-10 years (Males = 10, Females = 07), and 30 were above 10 years of age (Males = 20, Females =10). Twenty (31.25%) patients presented with DKA. Median age at presentation was 8.5 years (1-16 years). Median hospital stay was 6 (5-7) days, and median HbA1c at admission was 12.3% (11.35%-13.2%).

Insulin dose

We studied the maximum insulin dose required during admission and the insulin dose at discharge, and their correlation with different patient variables. The maximum insulin dose required during the hospital stay in individual patients ranged from 0.86 IU/kg/day to 1.7 IU/kg/day, with discharge doses ranging from 0.7 IU/kg/day to 1.2 IU/kg/day.

Insulin dose requirement and age

The maximum insulin requirement during hospitalization differed significantly among age groups (Kruskal-Wallis test, H = 8.72, p = 0.022), with children <5 years old requiring the highest doses. However, the insulin dose at discharge did not differ significantly across age groups (Kruskal-Wallis test, H = 3.35, p = 0.247) (Table 1).

**Table 1:** Insulin requirements in different age groups

Insulin requirement	Age groups			P value
	<5 y (N = 17)	6-10 y (N = 17)	>10 y (N = 30)	
Maximum requirement during hospital stay (IU/kg/day)	1.3 (1.3, 1.7)	1 (0.86, 1.1)	1 (0.9, 1.2)	0.022
At discharge (IU/kg/day)	1 (0.87, 1.3)	0.77 (0.7, 1.05)	0.83 (0.7, 1)	0.247

Insulin dose requirement and gender

Maximum insulin requirement during hospitalization was significantly higher in females than in males (Mann-Whitney U = 353.0, p = 0.027). However, the discharge insulin dose did not differ significantly between sexes (Mann-Whitney U = 451.5, p = 0.377) (Table 2).

**Table 2:** Insulin requirement based on gender

Insulin requirement	Male (N = 39)	Female (N = 25)	P value
Maximum requirement during hospital stay (IU/kg/day)	1 (0.85, 1.15)	1.2 (1, 1.5)	0.027
At discharge (IU/kg/day)	0.83 (0.7, 1)	0.83 (0.75, 1.3)	0.377

Insulin requirement and clinical presentation with DKA

**Figure 2:**
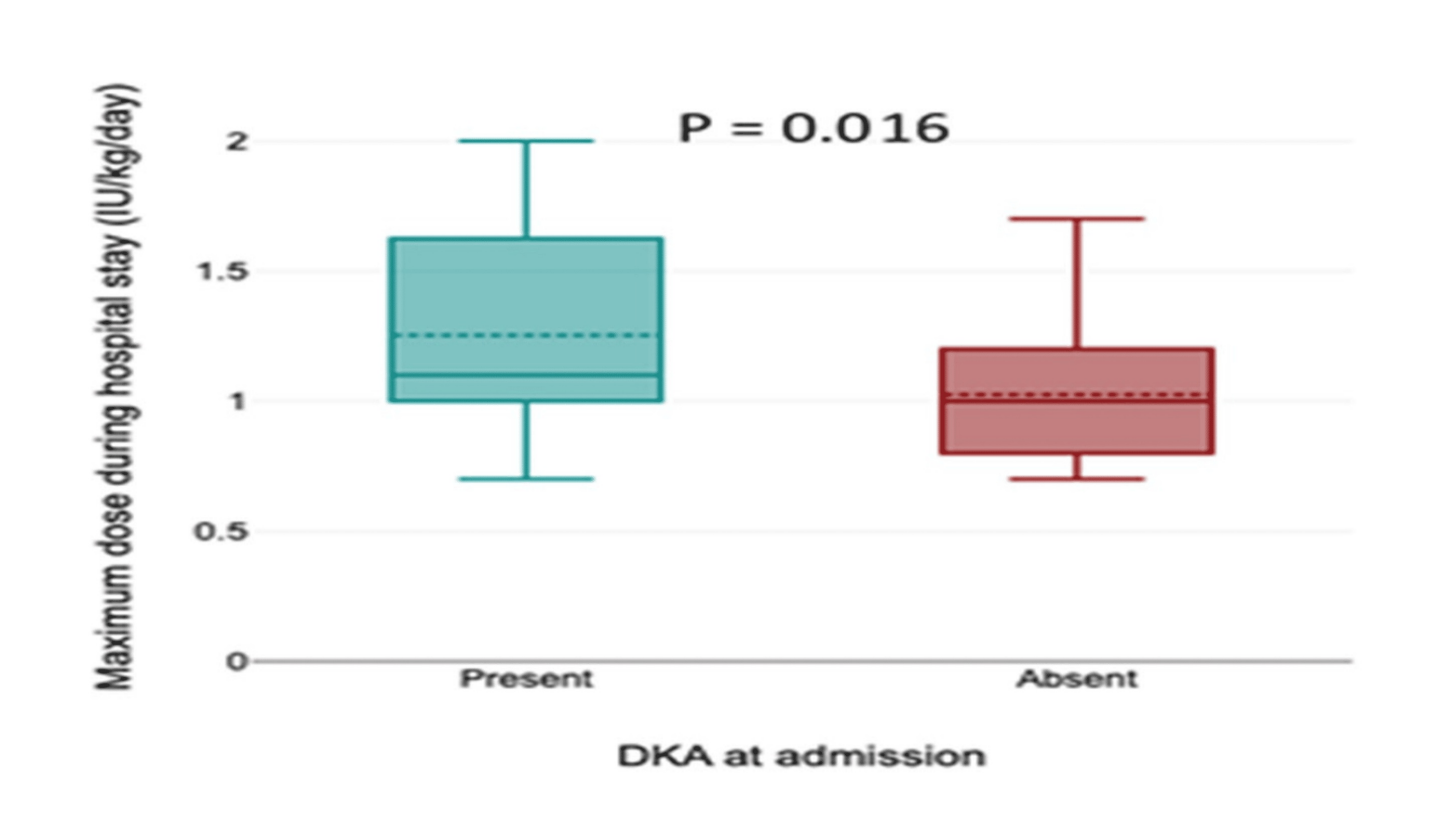
Insulin requirement based on clinical presentation DKA: diabetic ketoacidosis

Patients presenting with DKA required significantly higher maximum insulin doses during hospitalization (Mann-Whitney U = 297.5, p = 0.016). However, the discharge insulin dose did not differ significantly between patients with and without DKA (Mann-Whitney U = 361.0, p = 0.175) (Table 3, Figure 2).

**Table 3:** Insulin requirement based on clinical presentation DKA: diabetic ketoacidosis

Insulin requirement	DKA at presentation to the hospital		P value
	Present (N = 20)	Absent (N = 44)	
Maximum requirement during hospital stay (IU/kg/day)	1.1 (1, 1.63)	1 (0.8, 1.2)	0.016
At discharge (IU/kg/day)	0.86 (0.73, 1.3)	0.8 (0.66, 1)	0.175

Insulin requirement and comorbidities

**Figure 3:**
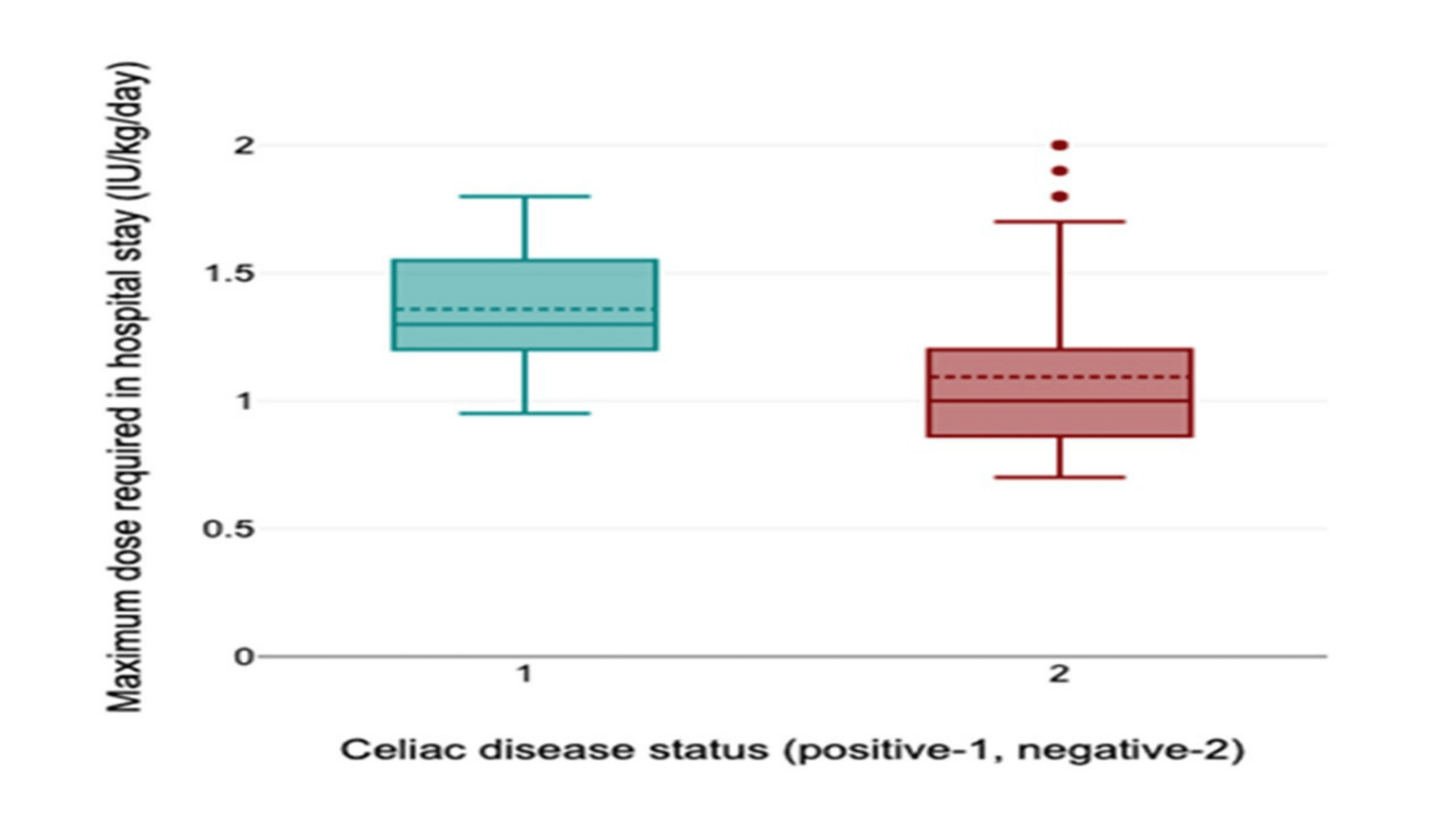
Insulin requirement in patients with and without celiac disease

Children with celiac disease had significantly higher maximum insulin requirement during hospitalization (Mann-Whitney U = 58.0, p = 0.040) and higher insulin dose at discharge (Mann-Whitney U = 80.0, p = 0.031) (Table 4, Figure 3). No significant difference in maximum insulin requirement and discharge insulin dose was noted among those having hypothyroidism as compared to euthyroid patients.

**Table 4:** Insulin requirement in T1DM children with celiac disease T1DM: type 1 diabetes mellitus

Insulin requirement	Celiac disease		P value
	Present (N = 5)	Absent (N = 59)	
Maximum requirement during hospital stay (IU/kg/day)	1.3 (1.2, 1.55)	1 (0.86, 1.2)	0.040
At discharge (IU/kg/day)	1 (1, 1.3)	0.8 (0.7, 1)	0.031

Insulin requirement and treatment protocol

There was no significant difference in maximum insulin requirement between children treated with basal-bolus and split-mix regimens (1.05 IU/Kg/day vs 1 IU/Kg/day, Mann-Whitney U = 297.5, p = 0.917). Similarly, discharge insulin dose did not differ significantly between the basal-bolus and split-mix regimens (0.87 IU/Kg/day vs 0.85 IU/Kg/day, Mann-Whitney U = 325.5, p = 0.843).

Linear regression analysis identified age, sex, presence of DKA, and maximum insulin dose required during hospitalization as significant predictors of insulin dose at discharge (R2 = 0.92, p < 0.001). The maximum insulin dose required during the hospital stay (β = 1.05) was the most significant predictor, followed by age (β = 0.15), absence of DKA (β = 0.08), and male sex (β = 0.06). This relationship can be expressed as:

Discharge insulin dose (IU/kg/day) = −0.28 + 0.06(M) + 0.15(age in years) − 0.08(DKA) + 1.05(maximum insulin dose during hospital stay)

Where:

M = 1 for males, 0 for females,

DKA = 1 if diabetic ketoacidosis was present at admission, 0 if absent.

## Discussion

In our study, we analyzed variations in discharge insulin dose and maximum insulin dose during admission among pediatric patients aged 18 years or less with T1DM. We attempted to correlate these with patient variables such as age, weight, sex, and duration of hospital stay, as well as comorbidities such as celiac disease and hypothyroidism.

To the best of our knowledge, limited data are available from developing country settings, particularly India, regarding daily insulin requirement among children. Also, individual insulin dose titration during admission can prolong hospital stay, and no consensus guidelines exist to date regarding insulin dose titration based on patient variables, as we mentioned earlier. Previously, Muller et al. found, in a retrospective observational study, that body size (total body weight and fat-free mass), HbA1C, and blood ketone concentration were significant predictors of optimal insulin total daily dose (p < 0.05) [2]. Caruthers et al. in 2019 concluded that there should be a reduction in the pre-emptive insulin daily dose at discharge for patients with newly diagnosed diabetes, ketosis-prone diabetes, metformin prescription, and HbA1c < 10% at presentation [7].

Also, Yazidi et al. found a negative correlation between age at diabetes onset and HbA1c (p=0.02). They did not find a significant relationship between the number of daily insulin injections and the mean HbA1c value. In their study, mean HbA1c was higher in patients with poor compliance to insulin therapy (11.1±3.3% vs. 8.9±2.4%, p < 0.0001) and adolescents vs adults (10.8±2.9% vs. 9.2±2.8%, p = 0.02), in those with less than three clinic visits per year (10.7±3.5% vs. 9.0±2.1%, p = 0.001), in subjects with lipohypertrophy (10.9±2.5% vs. 9.2±3.4%, p = 0.008) and those with known celiac disease (14.5±5.2% vs. 9.6±2.9%, p = 0.005)[8]. A Tanzania-based study by Noorani et al. in 2016 concluded that younger age and having the mother as the primary caregiver were associated with better glycemic control [9]. Most studies considered various patient variables affecting diabetes control, measured as HbA1c (%).

Limitations of the study

As the treatment allocation of the insulin regimen was not randomized and was based on affordability, this comparison should be interpreted very cautiously. Other limitations were: a single-centre design with a small sample size, a very small celiac subgroup, a mixed retrospective/prospective design, a lack of pubertal status/BMI/body composition data, and no long-term follow-up.

## Conclusions

Our study concluded that age, sex, presence of DKA, and the maximum insulin dose required during hospitalization are significant predictors of discharge insulin dose. Maximum insulin requirement during hospitalization, as well as discharge insulin dose, were significantly higher among children below 5 years of age, female patients, those presenting with DKA, and those having celiac disease. These findings provide valuable insights for refining insulin titration and expediting patient discharge. We emphasize conducting more such research in the future to explore correlations with additional patient variables, warranting larger sample sizes. Further studies are imperative in developing nations, given the limited data on pediatric insulin titration.

## References

[1] (1987). Diabetes Control and Complications Trial (DCCT): results of feasibility study. The DCCT Research Group. Diabetes Care.

[2] Muller M, Wheeler BJ, Blackwell M, Colas M, Reith DM, Medlicott NJ, Al-Sallami HS (2018). The influence of patient variables on insulin total daily dose in paediatric inpatients with new onset type 1 diabetes mellitus. J Diabetes Metab Disord.

[3] Mitsui Y, Kuroda A, Ishizu M (2022). Basal insulin requirement in patients with type 1 diabetes depends on the age and body mass index. J Diabetes Investig.

[4] Cengiz E, Danne T, Ahmad T (2022). ISPAD Clinical Practice Consensus Guidelines 2022: insulin treatment in children and adolescents with diabetes. Pediatr Diabetes.

[5] American Diabetes Association (2018). 8. Pharmacologic approaches to glycemic treatment: standards of medical care in diabetes-2018. Diabetes Care.

[6] Blonde L, Umpierrez GE, Reddy SS (2022). American Association of Clinical Endocrinology clinical practice guideline: developing a diabetes mellitus comprehensive care plan-2022 update. Endocr Pract.

[7] Carruthers D, Ismaily M, Vanderheiden A (2019). Determining insulin dose at the time of discharge in a high-risk population: is there room for improvement?. Endocr Pract.

[8] Yazidi M, Chihaoui M, Chaker F, Rjeb O, Slimane H (2016). Factors predicting glycemic control in type 1 diabetic patient. Open Med J.

[9] Noorani M, Ramaiya K, Manji K (2016). Glycaemic control in type 1 diabetes mellitus among children and adolescents in a resource limited setting in Dar es Salaam - Tanzania. BMC Endocr Disord.

